# Effect of Immediate Dentin Sealing on the Bonding Performance of Indirect Restorations: A Systematic Review

**DOI:** 10.3390/biomimetics9030182

**Published:** 2024-03-17

**Authors:** Fusun Ozer, Zeynep Batu Eken, Jessica Hao, Nuray Tuloglu, Markus B. Blatz

**Affiliations:** 1 School of Dental Medicine, Department of Preventive and Restorative Sciences, University of Pennsylvania, Philadelphia, PA 19104, USA; 2 Faculty of Dentistry, Department of Restorative Dentistry, Yeditepe University, Istanbul 34728, Turkey; 3 School of Dental Medicine, University of Pennsylvania, Philadelphia, PA 19104, USA; haoje@upenn.edu; 4 Faculty of Dentistry, Department of Pediatric Dentistry, University of Eskisehir Osmangazi, Eskisehir 26040, Turkey

**Keywords:** adhesive agents, bond strength, dental materials, immediate dentin sealing, indirect restorations, restorative dentistry, systematic review

## Abstract

The popular immediate dentin sealing (IDS) technique is used to improve the bond strength of indirect restorations. This systematic review assessed whether bond strength is affected by the type of aging conditions, bonding agents, flowable resin composites, impression materials, temporary materials, and/or resin cement used within the IDS procedure. A comprehensive database search of PubMed, Embase, Scopus, Ovid Medline, Web of Sciences, Cochrane Library, Dentistry & Oral Sciences Source, and ProQuest was carried out up to 30 January 2024 without publication year or language limitations. Only in vitro full-texts regarding the effect of IDS on bond strength were included, and the quality of their methods was assessed via a Risk of Bias (RoB) test. In total, 1023 pertinent studies were initially found, and 60 articles were selected for review after screening for the title, abstract, and full texts. IDS application improves the bond strength of indirect restorations to dentin and reduces the negative effects of temporary materials on the bond durability of final indirect restorations. Filled dentin bonding agents or combinations with flowable resin composite are preferred to protect the IDS layer from conditioning procedures.

## 1. Introduction

The traditional protocol for indirect esthetic restorations includes preparing the tooth, making an impression, and inserting a temporary restoration before fabricating and inserting the definitive restoration [1]. During the temporary phase, the prepared dentin is prone to contamination and collagen degradation by temporary cement or infiltration by oral bacteria. In addition, dentin tubules exposed during tooth preparation provide a potential pathway to the pulp, which may result in postoperative sensitivity and pulpal injury [2]. To mitigate these issues, studies as early as the 1990s suggested sealing freshly cut dentin surfaces with dentin bonding agents (DBA) prior to impression making [3,4]. This technique, most commonly known as “immediate dentin sealing (IDS)”, has also been referred to as “resin coating” [5,6], “prehybridization” [7], or “dual-bonding” [4]. 

Application and polymerization of DBAs through IDS can reduce the permeability of dentin by forming an interdiffusion layer, or hybrid layer, through the interpenetration of monomers into the hard tissues [8]. IDS provides many advantages, including tissue conservation, improved patient comfort, reduced bacterial contamination and marginal leakage, pulpal protection, and improved bond strength [2,8,9].

One of the most important reasons for supporting IDS is its claimed positive impact on the bond strength of definitive restorations [5,8]. Since bond strength is commonly considered a reliable indicator of the longevity of dental restorations [10], this positive effect can have strong implications on the lifespan of indirect bonded restorations such as composite/ceramic inlays, onlays, and veneers [8]. Freshly cut dentin is considered the ideal substrate for dentin bonding, but using the traditional protocol for indirect esthetic restorations may significantly reduce bond strength due to contaminations with various temporary cements [4,11,12]. The role of IDS in effectively preserving the state of the exposed tooth may contribute to the observed increased bond strength. 

Despite the generally positive impact of IDS on the bonding performance of indirect restorations, manipulating certain factors within the procedure can have drastic effects on indirect restoration adhesion. Since there is currently no consensus on which combination of factors achieves the highest bond strength, this systematic review analyzes the effectiveness of different materials used in IDS on bonding performance. Specifically, the available literature was compared based on the different types of DBAs, combinations with flowable resin-based composites (RBC), impression materials, temporary materials, resin cement, and/or restorative materials. This systematic review evaluated and compared in vitro studies regarding bonding performance after the use of IDS to analyze the differences and benefits of the techniques used across the studies. 

## 2. Materials and Methods

The authors carried out the present systematic review according to PRISMA guidelines [13]. The following PICOS framework was used: problem (P): bond strength of indirect restorations; intervention/indicator (I): IDS technique via differential aging conditions, DBAs, flowable RBC, impression materials, temporary materials, resin cement, and/or restorative materials; comparison (C): conventional/delayed dentin sealing (DDS) technique via differential aging conditions, DBAs, flowable RBC, impression materials, temporary materials, resin cement, and/or restorative materials; outcome (O): shear bond strength, microshear bond strength, microtensile bond strength, and tensile bond strength values; study design (S): in vitro studies. The research question is: which combination of materials in the IDS procedure may be optimal in achieving the highest bond strength? 

### 2.1. Literature Search Strategy

An exhaustive search of PubMed, Embase, Scopus, Ovid Medline, Web of Sciences, Cochrane Library, and the Dentistry & Oral Sciences Source was conducted, and full texts were collected until 30 January 2024. No restrictions were set on the language or year of the study, and the grey literature database ProQuest was searched in a similar manner. Furthermore, a database search for free terms in the titles and abstracts was conducted separately by two authors (J.H. and Z.B.E.) using the keywords: (“Immediate Dentin Sealing” OR “resin coating” OR “pre-hybridization” OR “prehybridization” OR “dual-bonding”) AND (“bond strength” OR “bonding strength”). (Table 1) The search strategy has been adapted to the other databases. 

In addition to a free term search, a controlled vocabulary search was also conducted. However, it was discovered that subject headings similar to the keywords used were not present in the databases. Thus, the authors attempted other methods of searching, such as a reverse strategy that utilized the subject headings in articles selected from the keyword search. However, any shared subject headings between the keyword-search articles were too general and resulted in exceedingly broad searches. Finally, the authors attempted another search method, combining subject headings with keywords and/or subheadings to decrease the number of results. However, the results remained too general to be incorporated into the screening process. Therefore, with all methods exhausted, the authors decided that this present systematic review would not employ subject headings in its searches. 

### 2.2. Eligibility Criteria

Full-text studies that pertained to the effect of IDS on the bond strength of indirect restorations to dentin and included a control group with conventional/DDS technique were added to this systematic review. Article abstracts, short communications, case reports, observational studies, reviews, and publications that pertained to other properties of IDS were excluded from the review. 

### 2.3. Screening and Selection

The titles and abstracts of the collected studies were examined by three of the reviewers (N.T., J.H., Z.B.E.), who discussed their differences in opinions until a consensus was reached for the articles that fit the inclusion criteria. Full texts of each of these titles were then recovered and assessed for inclusion and detailed assessment of the experimental conditions. Finally, the reviewers considered the references from the chosen articles and determined the potential eligibility of articles in the references. Any disagreements between the three reviewers were settled by consulting a fourth reviewer (M.B.B.).

### 2.4. Data Extraction

Data was obtained from the chosen full texts and compiled on an Excel sheet by three of the reviewers (F.O., J.H., Z.B.E.). The obtained data included author names, publication year, tooth type, sample size, test method, and specific methodologies (adhesive agent, resin composite, restoration material, temporary material, conditioning method, luting cement, and aging).

### 2.5. Risk of Bias (RoB) Assessment

Two reviewers (J.H. and Z.B.E.) used a Risk of Bias (RoB) test to measure the methodological quality of the selected articles. Each article was evaluated based on (I) randomizing the teeth, (II) using materials in accordance with the manufacturer’s instructions, (III) administering treatments with the same operator, (IV) description of the sample size calculation, (V) standardized sample preparation, (VI) blinding of the testing machine operator, and (VII) failure mode analysis. 

Since most of the literature evaluated in the present study is in vitro experiments, the Cochrane RoB tool was unable to be used since it was designed for the evaluation of clinical trials. Therefore, the authors adapted a RoB methodology used in a similar review paper [14]. If the authors of the study stated the parameter, the article was given a ‘‘Y’’ (yes) on that specific parameter; if there was no information, the article then received an ‘‘N’’ (no). Articles that reported a “Y” in 1–3 items were classified as having high RoB, 4–5 items as medium RoB, and 6–7 items as low RoB. 

### 2.6. Inter-Rater Reliability (IRR)

Since the RoB assessment was performed by two reviewers independently of one another, an inter-rater reliability (IRR) test needs to be performed to determine the degree of difference between the two reviewers’ designations. The IRR test was conducted using the kappa calculator on SPSS Statistics Version 19.0 (IBM, Armonk, NY, USA) following the procedure outlined in Hao et al. [15] and McHugh [16]. This test calculated the percent user agreement by dividing the number of articles with the same RoB from both reviewers by the total number of articles. To run a Cohen’s Kappa test, which requires the difference between the two author’s designations, “Y” was converted to 1, and “N” was converted to 0, and the resulting kappa values are reported. In order to find a reliable percentage of data, the reviewers squared the kappa values from each of the parameters. Finally, using the percentages, the reviewers were able to characterize a level of agreement for each of the parameters [16].

## 3. Results

### 3.1. Search and Selection

**Figure 1:**
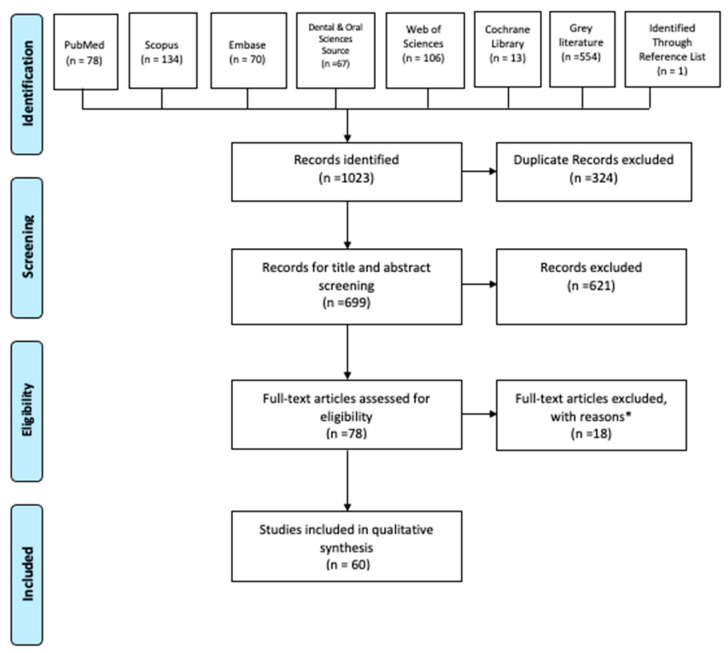
The Prisma flow diagram. *: 12 did not include a control (DDS) group, 6 were unable to be accessed in English.

Altogether, the database, grey literature, and reference search showed 1023 pertinent articles. The flowchart of the article selection procedure, according to the PRISMA guidelines, is presented in Figure 1. After duplicate removal, the reviewers considered 699 records for their titles and abstracts. A total of 621 studies were eliminated for not adhering to the eligibility criteria, and the full texts of 78 articles were assessed. Of the 78 articles saved for more comprehensive analysis, 12 were eliminated for not including a conventional/DDS group and 6 for not being in English. One study found during the manual search in the references of the selected articles was included. Finally, 60 studies fulfilled all the selection criteria initially outlined by the reviewers and were included. The studies that had a control group of DDS technique that mimicked the clinical scenario of conventional technique or an uncoated surface were included.

Table 2 describes details of the included studies, such as publication year, type of tooth, IDS material, restoration material, temporary material, conditioning method, sample size, test method, and aging. Among bond strength methodologies used by the included studies, 37 studies evaluated microtensile bond strength to dentin [17,18,19,20,21,22,23,24,25,26,27,28,29,30,31,32,33,34,35,36,37,38,39,40,41,42,43,44,45,46,47,48,49,50,51,52,53], 15 evaluated shear bond strength to dentin [54,55,56,57,58,59,60,61,62,63,64,65,66,67,68], 3 evaluated microshear bond strength to dentin [69,70,71], and 5 evaluated tensile bond strength to dentin [72,73,74,75,76]. The majority of the studies used human molar and premolar teeth, while eight studies used bovine teeth for the bond strength test [50,69,70,71,72,73,74,75]. The majority of the studies conducted bond strength tests after 24 h. Nine studies [19,26,34,45,53,54,57,63,68] used thermocycling, six studies [30,38,41,44,51,52] used cyclic loading for aging, one study [32] used both thermocycling and cyclic loading, and six studies [28,39,42,71,72,74] stored in water for different periods of time. A total of 15 studies compared the effect of etch-and-rinse (ER) and self-etch (SE) strategies on the bond strength of IDS [18,19,20,29,39,46,53,56,58,60,63,69,73]. Among the studies that used temporary filling materials, 12 studies [20,21,23,24,27,28,31,32,49,73,75,76] used water-setting temporary materials, 8 studies [17,18,19,36,46,48,55,64] used light-cure temporary materials, and 12 studies [25,37,40,42,44,50,54,57,59,63,69,70] used temporary cement. In nine studies [19,22,24,26,27,46,55,59,76], the impressions of the cavities were taken.

### 3.2. Risk of Bias (RoB) Test of the Studies in the Systematic Review

After analyzing the 60 articles for their RoB, both authors gave the majority of papers “N” in the “(III) single operator”, (IV) sample size, and “(V) blinding of operator” criteria for lack of information. A total of 25 studies showed high RoB levels, 34 studies showed medium, and 1 showed low (Table 3).

### 3.3. Inter-Rater Reliability Results

Results from the IRR tests for each RoB parameter are shown in Table 4. Overall, the RoB parameters are above 95.00% in the percent user agreement, with the average being 99.047%. Based on Cohen’s Kappa Test, the average percent of reliable data is 93.155%, which correlates to an almost perfect reliability. The average kappa value is 0.962. Parameter II, or using materials in accordance with the manufacturer’s instructions, had an especially weak agreement level since the articles were variable in their degree of explanation. While the adherence to the manufacturer’s instructions was explicitly stated in some, others mentioned the criteria vaguely in a table, thus resulting in inconsistencies between the two reviewers. However, all discrepancies were resolved by the two reviewers after discussion. 

## 4. Discussion

Despite IDS’s prevalence, procedural variability can affect indirect restoration adhesion. This variability alters bond strength between different interfaces like the restorative material and resin cement, resin cement and IDS layer, or IDS layer and dentin. The literature search of studies exploring IDS’s effect on bond strength revealed heterogeneity in experimental methods and conditions. Therefore, in vitro bond strength studies were compared based on DBAs, flowable RBCs, impression materials, temporary materials, and/or resin cement. The control groups of the evaluated studies included dentin surfaces that were treated according to the DDS technique with the application of temporary and/or impression material, stored in water for a certain time, or uncoated surfaces without mimicking the conventional procedure. However, the increased use of CAD/CAM systems in recent years reduced the need for temporary materials and impressions. Therefore, it became possible to bond the indirect restorations immediately. Consequently, the comparison of bonding to an uncoated surface and IDS is as important as the comparison of conventional techniques to IDS. Overall, the literature supported IDS’s ability to improve bond strength. A few showed negative or no impact [36,58,59,67], while several showed benefits contingents upon the resin cement [34,35,43,45,57], DBA [39,46,56], or flowable RBC [23,32,69].

### 4.1. Effect of Dentin Bonding Agents and Flowable Resin-based Composites

Since Pashley et al. [3] introduced dentin sealing in 1992, many DBAs have been used to seal freshly cut dentin. In 2005, Magne et al. [8] pioneered IDS by using filled DBAs (Optibond FL (OFL); Kerr Corporation, Orange, CA, USA) or combining unfilled DBAs and flowable RBC. Unfilled DBAs form thinner layers, and surface cleaning procedures may destroy the hybrid layer and re-expose dentin [8]. Applying additional flowable RBC reduces dentin exposure risk, mitigates stress on the interface, and eliminates the oxygen inhibition layer by sealing DBA. Similarly, the original resin coating technique involves two-step self-etching with flowable RBC [5]. 

When two gold standard DBAs—3-step ER (OFL) and 2-step SE (Clearfil SE Bond; Kuraray Medical Inc., Tokyo, Japan)—were compared, OFL showed higher or similar bond strength [18,39,46,63]. In addition, 2-step SE (Clearfil SE Bond) showed higher bond strength than 2-step ER, regardless of using flowable RBC [20,56]. However, few studies that investigated different DBAs, including 3-step ER, 2-step ER, 2-step SE, and 1-step SE without flowable RBC, exhibited no benefit from IDS [36,58,59]. The composition of the adhesives and the filler content may play a role in the different performance of the adhesives. Optimally filled adhesives showed increased mechanical properties of the adhesive layer and increased bond strength [77,78]. An adhesive layer with increased mechanical properties may help the stress distribution and act as a shock absorber. However, high filler size and content also lead to high viscosity and reduced penetration of the adhesive into the dentin [79]. Furthermore, the high viscosity of the filled adhesives would lead to pooling at the margins. 

Half of the investigated studies combined DBA with flowable RBC, which enhanced bond strength more than DBA alone [20,23,32,46,69]. Filled OFL improved bond strength compared to DDS, even without flowable RBC. Unfilled/lightly filled DBAs should be applied with flowable RBC in the “reinforced IDS” approach to improve μTBS to dentin [46].

Adding flowable RBC or extra adhesive layers similarly affected bond strength [42,63]. However, the double application of all-in-one adhesives improved resin coating bond strength [31,75]. One study investigated the effect of IDS form and thickness applied by universal adhesive and a flowable RBC on intra-cavity μTBS. Thicker IDS layers may act as a stress-breaker under cyclic load stress, and moderate and thick IDS layers’ bond strength was higher than thin or no IDS [41]. Therefore, combining DBA with flowable RBC is recommended. Akehashi et al. [43] compared dual-cure resin cement as the IDS material with flowable RBC and reported that combining two-step SE adhesive with dual-cure resin cement as IDS showed the closest results to the bond strength of direct restorations. 

### 4.2. Effect of Impression Materials

Following IDS application, impression materials may interact with the outer resin layer [4], which is unpolymerized due to oxygen inhibition [8,80,81], yielding an unpolymerized layer of impression or impression material adhesion and tearing on the IDS surface [9,82]. Eliminating oxygen-inhibition layers (OIL) is necessary to prevent interference with impression material setting and temporary restorative material adherence [9]. Cleaning with an alcohol-soaked cotton pellet, pumice, or application of glycerin gel with additional polymerization are accepted methods to eliminate OIL [9,83,84]. Although it is possible to obtain successful impressions with vinyl polysiloxane following air blocking or pumicing of the sealed surface, there is the risk of faulty impressions due to adhesion and tearing with polyether impression material [9,85]. In several studies, cavity impressions were taken due to preparation type or to simulate clinical practice. However, the included studies did not evaluate the effect of impression materials on bond strength with IDS.

### 4.3. Effect of Temporary Materials and Conditioning Methods

Since contaminating dentin with temporary materials reduces adhesion [25,37,54], IDS should be applied before temporary restorations. However, temporary cement remnants may also contaminate the IDS surface and decrease bond strength to sealed dentin. Most studies showed IDS’s benefits, regardless of temporary materials or cleaning methods. Sealed dentin surfaces can bind resin-based temporary materials due to the OIL. Therefore, retrieving and removing temporary materials may be difficult [6,18]. Isolating sealed dentin with a glycerin gel [36] or petroleum jelly [18] can inhibit the interaction with resin-based temporary materials. Removal of non-eugenol temporary cement with the excavator and cleaning with alcohol may not be enough to remove cement remnants from the IDS surface and lead to a reduction in bond strength [50]. Therefore, the conditioning method is important to remove temporary cement’s harmful effects. 

Different conditioning methods, such as airborne-particle abrasion with aluminum oxide, etching with phosphoric acid, polishing with pumice, and tribochemical silica coating, were used beneficially with IDS. With the tribochemical silica coating method, silica particles are deposited on the surface, which leads to an increase in the surface [86]. However, three studies compared the effects of different conditioning methods on the IDS surface [42,59,63]. Cleaning with pumice only, or with additional tribochemical silica coating of zinc-carboxylate cement [42], or temporary zinc-oxide luting cement [63] did not affect bond strength. However, the conditioning method depends on the type of IDS material type used. Tribochemical silica coating or sandblasting may remove a thin IDS layer and result in decreased bond strength [42]. After conditioning, the dentin exposure risk may be reduced with filled adhesives or flowable RBC [42,87]. Therefore, a thick IDS layer created with an extra adhesive layer or flowable composite when using silica-coating conditioning is recommended by the authors in clinical practice [63]. 

### 4.4. Effect of Resin Cement

Self-adhesive (SA) resin cement has gained popularity due to the reduced postoperative sensitivity and application simplicity [88] but has lower bond strength than conventional resin cement [89]. Among studies indicating IDS’s dependence on resin cement types, two [43,45] compared different conventional SE resin cement and three [34,35,57] compared conventional resin cement with SA resin cement. Others [37,55,62] showed IDS improved bond strength, regardless of the cement. Nevertheless, curing through restorative materials characterized by different translucency [90] may influence the overall cement conversion, thus possibly affecting bond strength to the dentin surface. In addition to resin cement, IDS improved the bond strength of dual-curing compomer cement [62] and resin composite as a luting material [55,76].

The remaining OIL on the resin coating following light curing may help the resin cement bond to the coating materials [91]. However, this unpolymerized layer should be removed to prevent interference with impression materials. Therefore, resin-coated surface pretreatment is important to improve the adhesion with resin cement. 

### 4.5. Bonding Efficiency to Different Restorative Materials and Preparation Types

A higher C-factor in inlay cavities causes polymerization contraction stresses on bond strength and reduces internal adaptation [45]. However, IDS was found beneficial for Class II inlay [19,24,45], Class I inlay [23,25,26], indirect Class V restoration [40], onlay [38,41] overlay [61], and crown restoration bond strength [22,30,44,51], in addition to flat dentin surfaces. IDS application on crown preparations is more technique-sensitive due to the preparation deformation risk and adhesive pooling on the preparation shoulder. 

Regarding the restorative material, IDS improved bond strength to ceramic [41,44,51,61], zirconia [62], metal [60], and resin composite materials. Conventional indirect restorations involve complicated procedure steps, while CAD/CAM restorations have several advantages. Ishii et al. [38] compared the intra-cavity bond strength of different CAD/CAM blocks with and without IDS. IDS improved the bond strength of Lava Ultimate and Vitablocks Mark II. However, resin composite blocks (Lava Ultimate; 3M ESPE; St. Paul, MN, USA, and Vita Enamic; VITA Zahnfabrik, Bad Säckingen, Germany) showed significantly higher bond strength than glass–ceramic blocks (Vitablocks Mark II; VITA Zahnfabrik, Bad Säckingen, Germany), regardless of IDS. The resin cement and surface treatments of the restorative materials play important roles in the interface. 

### 4.6. Effect of Aging Conditions

Overall, IDS improved bond strength after aging; however, some studies that evaluated IDS stability over time [28,30,39,42,72] showed contradictory results regarding IDS’s effect on interface durability. Although IDS may initially improve bond strength depending on the DBA, it did not prevent decreasing bond strength after water storage for 3 months [39] and 6 months [49]. Another study reported the adhesive interface with IDS was stable after water storage for 6 months [42]. It should be noted that the aging method used in some of the studies [19,26,54,57] included 1000–1500 thermocycles, which is not sufficient for aging standards [92,93]. Therefore, there is still a need for long-term durability studies. 

Magne et al. [9] recommended sealing dentin surfaces with a DBA immediately after tooth preparation for indirect restorations. They confirmed that the bond strengths were not changed by up to 12 weeks of elapsed time before the cementation of permanent restorations.

### 4.7. Study Limitations

Quantitative evaluation through meta-analysis could not be performed in this present review due to the heterogeneity of the evaluated studies’ methods. Additionally, owing to the lack of standardized criteria for assessing the RoB and in vitro study quality, a previous study’s methodology was adopted. Therefore, more recent in vitro and in vivo studies are needed to better analyze laboratory and clinical correlation data. 

## 5. Conclusions

The evaluation of the included studies shows that IDS application improves the bond strength of indirect restorations to dentin and reduces the negative effects of temporary materials on the performance and the long-term durability of final indirect restorations. Filled DBAs or combinations with flowable RBCs are preferred to protect the IDS layer from conditioning procedures. In order to evaluate the potential relationship between bond strength data and clinical outcomes of the studies, more in vitro and clinical studies are needed.

## Figures and Tables

**Table 1:** Search strategy used in PubMed.

#1	“Immediate Dentin Sealing” OR “resin coating” OR “pre-hybridization” OR “prehybridization” OR “dual-bonding”
#2	“bond strength” OR “bonding strength”
	#1 and #2

**Table 2:** Summary of the studies included in the systematic review.

Author, Year	Type of Tooth	IDS Material (DBA + Resin Composite)	Restoration Material	Temporary Material	Cleaning Method of IDS Surface	Luting Cement	Aging	Specimen Number per Group	Test Method
Kitasako et al. [72] (2002)	Bovine	Clapearl bonding agent + Protect Liner F	Clearfil CR Inlay (indirect resin composite)	-	-	-Clapearl DC -Panavia 21 -Super Bond C&B	37 °C tap water for 1 day, 6 months, 1 year and 3 years	10	TBS
Nikaido et al. [73] (2003)	Bovine	-Clearfil SE (SE) + Protect Liner F -Unifil Bond (SE) + Protect Liner F -One-Up Bond (SE) + Protect Liner F -Single Bond (ER) + Protect Liner F	Estenia (indirect resin composite)	Cavit-G	Cotton pellet moistened with 70% ethanol, etched with 37% phosphoric acid	-Panavia F -Link Max -Bistite II -Rely-X	Water at 37 °C for 1 day	10	TBS
Jayasooriya et al. [20] (2003)	Human (premolar)	-Clearfil SE Bond (SE) -Clearfil SE Bond (SE) + Protect Liner F -Single Bond (ER) -Single Bond (ER) + Protect Liner F	-Estenia (indirect resin composite) -Clearfil AP-X (direct resin composite)	Cavit-G	Cotton pellet moistened ethanol, etched with 37% phosphoric acid	Panavia F	Water at 37 °C for 24 h	5	µTBS
Nikaido et al. [21] (2003)	Human (molar)	RZ-II (SE)-experimental	-Metafil C (direct resin composite) -New Metacolor Infis (indirect resin composite)	Cavit-G	Coton pellet soaked in alcohol, 10% citric acid, and 3% ferric chloride for 10 s	Chemiace II	Water for 1 day	4	µTBS
Magne et al. [17] (2005)	Human (molar)	Optibond FL (ER)	Z 100 (direct resin composite)	Tempfil Inlay	Airborne-particle abrasion	-	Distilled water at room temperature for 24 h	5	µTBS
Islam et al. [22] (2006)	Human (lower first molar)	Hybrid Bond (SE)	Estenia (indirect resin composite)	-	10% citric acid and 3% ferric chloride for 10 s	Chemiace II	Water at 37 °C for 24 h	5	µTBS
Duarte et al. [74] (2006)	Bovine (lower incisors)	Clearfil liner Bond 2V (SE) + Protect Liner F	Z 100 (indirect resin composite restoration)	-	-	Panavia F	Distilled water at 37 °C for 10 mins or 24 h or solution of deionized water and 0.4% sodium azide at 37 °C for 12 months	10	TBS
Okuda et al. [23] (2007)	Human (molar)	-Clearfil Protect Bond (SE) -Clearfil Protect Bond (SE) + Protect Liner F	-Estenia (indirect resin composite) -Clearfil AP-X (direct resin composite)	Cavit-G	Cotton pellet soaked in ethanol, 37% phosphoric acid for 10 s	Panavia F	Distilled water at 37 °C for 24 h	3	µTBS
Sultana et al. [24] (2007)	Human (third molar)	Clearfil SE Bond (SE) + Protect Liner F	Estenia (indirect resin composite)	Cavit-G	Coton pellet soaked in alcohol, 37% phosphoric acid for 10 s	Panavia F 2.0	Water at 37 °C for 24 h	11	µTBS
Frankenberger et al. [25] (2007)	Human (third molar)	-XP Bond (ER) -XP Bond (ER) + X-Flow -Syntac (ER) -Syntac (ER) + X-Flow -Optibond FL (ER) -Optibond FL (ER) + X-Flow	Clearfil AP-X (indirect resin composite restoration)	-TempBond -TempBond NE	-Scaler -Prophypearls -Clinpro powder	Calibra	Distilled water at 37 °C for 24 h	3	µTBS
Magne et al. [18] (2007)	Human (molar)	-Optibond FL (ER) -SE bond (SE)	Z 100 (direct resin composite)	Tempfil Inlay	Airborne-particle abrasion	-	Distilled water at room temperature for 24 h	5	µTBS
de Andrade et al. [26] (2007)	Human (molar)	-Single Bond (ER) -Single Bond (ER) + Protect Liner F	Targis Dentin-220 (indirect resin composite)	-	-	Rely X ARC	1200 thermal cycles	6–15 sticks	µTBS
Erkut et al. [54] (2007)	Human (molar)	-Single Bond (ER) -One Step (SE)	-	-RelyX Temp NE -RelyX Temp E	Pumice	-RelyX ARC -Duo Link	1000 thermal cycles, tap water at room temperature for one week	10	SBS
Ariyoshi et al. [27] (2008)	Human (molar)	-Clearfil SE Bond (SE) -Clearfil SE Bond (SE) + Clearfil Flow FX	Clearfil DC Core Automix (indirect resin composite core)	Caviton	Cotton pellet containing ethanol for 10 s	-Panavia F 2.0 -Clearfil DC Core Automix	Water at 37 °C for 24 h	5	µTBS
Nikaido et al. [28] (2008)	Human (molar)	-Clearfil SE Bond (SE) + Protect Liner F -Clearfil SE Bond (SE) + Ionosit MicroSpand	Estenia (indirect resin composite)	Cavit-G	-	Panavia F	37 °C water for 1 day, 6 months, or 1 year	10 sticks	µTBS
Santos-Daroz et al. [69] (2008)	Bovine (incisor)	-Single Bond (ER) -One-Up Bond F (SE) -Xeno III (SE) -AdheSE (SE) -Clearfil Protect Bond (SE) -Tyrian SPE/One-Step Plus SPE (SE) -Unifil Bond (SE) + Protect Liner F	-	Temp Bond NE	-	Panavia F	Water at 37 °C for 24 h	8	µSBS
Kameyama et al. [29] (2009)	Human (molar)	-UniFil Bond (SE) + UniFil Flow -Adper Single Bond (ER) + UniFil Flow	Gradia (indirect resin composite restoration)	-	Alcohol cotton pellet	Linkmax	Water at 37 °C for 24 h	4	µTBS
Kitayama et al. [30] (2009)	Human (lower third molar)	Clearfil Tri-S Bond (SE)	CEREC-Blocs (feldspathic ceramic)	-	Cotton pellet soaked in isopropyl alcohol	Clearfil Esthetic Cement	250,000 cycles of mechanical loading or storage in distilled water at 37 °C for 28 h	7	µTBS
Duarte et al. [19] (2009)	Human (third molar)	-Adper Single Bond (ER) -Adper Prompt L-pop (SE)	Targis Ceromer system	Fermit	Pumice and water	RelyX ARC	1000 thermal cycles	5	µTBS
Lee and Park [55] (2009)	Human (premolar)	AdheSe (SE)	Tescera ATL system (indirect resin composite)	Fermit	-	-Duo-Link -Filtek Z250	100% humidity at 37 °C for one day	15	SBS
Takahashi et al. [31] (2010)	Human (molar)	Tokuyama Bond Force (SE) (single and double layer)	Pearleste (indirect resin composite)	Caviton	Alcohol-soaked cotton pellets for 10 s, etching with 38% phosphoric acid	Bistite II	Water at 37 °C for 24 h	3	µTBS
Takahashi et al. [75] (2010)	Bovine (incisor)	-G-Bond (SE) -Clearfil Tri-S Bond (SE) -Tokuyama Bond Force (SE) -Hybrid-Coat (SE) (single and double layer)	Pearleste (indirect resin composite)	Caviton	Alcohol-soaked cotton pellets for 10 s, etching with 37% phosphoric acid	-Link Max -Clearfil Esthetic Cement -Bistite II -Chemiace II	Water at 37 °C for 24 h	10	TBS
Feitosa et al. [32] (2010)	Human (third molar)	-Clearfil S3 (SE) -Clearfil S3 (SE) + Clearfil Protect Liner -Clearfil SE Bond (SE) -Clearfil SE Bond (SE) + Clearfil Protect Liner	Sinfony (indirect resin composite)	Cavit	Pumice stone and water	Panavia F	1500 thermal cycles and 200,000 cyclic loading	5	µTBS
Choi and Cho [56] (2010)	Human (molar)	-Clearfil SE Bond (SE) -Adapter Single Bond 2 (ER)	Porcelain	-	-	Variolink II	Distilled water at 37 °C for 24 h	10	SBS
Hassan et al. [60] (2011)	Human (premolar)	-Clearfil SE Bond (SE) -Syntac® Sprint (ER)	Metal disc (Cobalt Chromium alloy)	-	-	-Panavia F -Variolink II	100% relative humidity at 37 °C for 24 h	12	SBS
Sailer et al. [57] (2012)	Human (molar)	Clearfil SE Bond (SE)	-	Freegenol	Abrasive fluoride-free polishing paste in combination with rubber cup	-RelyX Unicem -Variolink II -Panavia 21	Water storage at 37 °C for 24 h or 1500 thermal cycles or water storage at room temperature for 1 h	12	SBS
Dalby et al. [58] (2012)	Human (third molar)	-Optibond FL (ER) -Single bond (ER) -One Coat Bond (SE) -Go! (SE)	Authentic (Glass ceramic)	-	-	RelyX Unicem	Distilled water at room temperature for one week	16	SBS
Falkensammer et al. [59] (2014)	Human (premolar)	AdheSe (SE)	Vitablocks Mark II (felspathic ceramic blocks)	Temp Bond NE	Pumice, airborne-particle abrasion combined with silicoated aluminum oxide, glycine and calcium carbonate powder	Variolink II	Saline solution at 37 °C for 24 h	11	SBS
Duque [33] (2014)	Human (third molar)	-OptiBond FL (ER) -Optibond Solo Plus (ER)	Gradia (indirect resin composite)		-	RelyX Luting Plus	-	10	µTBS
Giannini et al. [34] (2015)	Human (third molar)	Clearfil SE Bond (SE) + Clearfil Majesty Flow	AP-X (indirect resin composite restoration)		-	-RelyX Unicem -RelyX Unicem 2 -Clearfil SA Cement -G-Cem -Panavia F 2.0	5000 thermocycles	5	µTBS
Santana et al. [35] (2016)	Human (third molar)	Clearfil SE Bond (SE)	Filtek Z250 (indirect resin composite restoration)	-	Airborne-particle abrasion	-RelyZ Unicem -Clearfil SA Luting -RelyX ARC -Panavia F	Distilled water for 24 h	5	µTBS
da Silva et al. [36] (2016)	Human (molar)	Adper ScotchBond Multipurpose (ER)	Z350 XT (direct resin composite)	-Dycal -Temp bond NE -Clip F	Pumice	-	Distilled water at 37 °C for 24 h	8	µTBS
Brigagão et al. [37] (2017)	Human (third molar)	Scotchbond Universal (SE and ER)	Z 100 (indirect resin composite restoration)	RelyX Temp	Rotary brush with pumice	-RelyX ARC -RelyX U200	Distilled water at 37 °C for 7 days	5	µTBS
Ishii et al. [38] (2017)	Human (mandibular first molar)	Scotchbond Universal (SE) + Filtek Supreme Ultra Flowable	-Lava Ultimate (indirect resin composite) -VITA ENAMIC (hybrid) -VITABLOCS Mark II (feldspathic ceramic)	-	Etching	RelyX Ultimate	Cyclic loading for 3 × 105 cycles	4	µTBS
Ferreira-Filho et al. [39] (2018)	Human (third molar)	-Xeno V (SE) -Clearfil SE Bond (SE) -XP Bond (ER) -Optibond FL (ER)	Filtek Z250 (indirect resin composite restoration)	-	-	RelyX Unicem	7 days or 3 months water storage at 37 °C	6	µTBS
Hironaka et al. [40] (2018)	Human (third molar)	Clearfil SE Bond 2 (SE) + Protect Liner F	Filtek Z250 (indirect resin composite restoration)	Temp Bond NE	Pumice and water	Panavia F 2.0	Artificial saliva at 37 °C for 24 h	10	µTBS
Murata et al. [41] (2018)	Human (maxillary first molar)	Scotchbond Universal (SE) + Filtek Supreme Ultra Flowable Restorative	VITABLOCS Mark II (feldspathic ceramic block)	-	-	Panavia V5	Cyclic loading for 3 × 105 cycles	8	µTBS
Reboul et al. [61] (2018)	Human (mandibular third molar)	OptiBond FL (ER)	Suprinity block (glass ceramic)	-	-	-Panavia V5 -Heated resin composite	Distilled water at room temperature for 7 days	10	SBS
Rigos et al. [62] (2019)	Human (third molar)	OptiBond FL (ER)	BruxZir (Monolithic zirconia block)	-	-	-Panavia F2.0 -PermaCem Dual Smartmix	Distilled water at 37 °C for 24 h	14–15	SBS
van den Breemer et al. [42] (2019)	Human (molar)	-OptiBond FL (ER) (1 and 2 layers) - OptiBond FL+Grand IO Flow	Enamel plus HFO (direct resin composite)	Durelon	-Pumice -Pumice + silica coating (Cojet)	-	1 week or 6 months of storage	24 sticks	µTBS
van den Breemer et al. [63] (2019)	Human (molar)	-Clearfil SE Bond (SE) (1 and 2 layers -OptiBond FL (ER) (1 and 2 layers) - Clearfil SE Bond + Grandio Flow - OptiBond FL + Grandio Flow	-	TempBond NE	-Pumice -Pumice + silica coating (Cojet)	Variolink II	10,000 thermocycles	10	SBS
Akehashi et al. [43] (2019)	Human (molar)	Clearfil SE Bond 2 (SE) + -Clearfil Protect Liner F -Clearfil Majesty LV -Panavia V5 (DC/LC)	-Estenia C&B (indirect resin composite) -Clearfil AP-X (direct resin composite)	-	-	-Panavia V5 -Panavia F2.0	Distilled water at 37 °C for 24 h	4	µTBS
Hayashi et al. [44] (2019)	Human (mandibular premolar)	Clearfil Universal Bond Quick (SE) + Clearfil Majesty ES Flow	VITABLOCS Mark II (feldspathic ceramic block)	TempBond NE	Polishing brush underwater	Panavia V5	Cyclic loading for 3 × 105 cycles	15 sticks	µTBS
Sag et al. [64] (2020)	Human (molar)	Clearfil SE Bond (SE) + Filtek Ultimate Flowable	-Lava Ultimate (Resin nano CAD-CAM block) -Solidex (indirect resin composite)	DiaTemp	-	-RelyX Unicem -RelyX Ultimate Clicker	-	10	SBS
Rozan et al. [45] (2020)	Human (third molar)	-G-Premio Bond (SE) -Clearfil SE Bond 2 (SE) + Clearfil Majesty ES Flow	Cerasmart (resin CAD/CAM block)	-	-	-RelyX Ultimate -G-CEM LinkForce -Panavia V5	5000 thermocycles	8	µTBS
Cesca et al. [76] (2020)	Human (maxillary central incisor, canine, and premolar)	Syntac (ER)	Tetric Ceram (direct and indirect resin composite restoration)	Cavit	Air-abrasion	-Preheated Tetric Ceram (resin composite) -Variolink II	Distilled water for 1 week	10	TBS
Carvalho et al. [46] (2021)	Human (third molar)	-Optibond FL (ER) -Scotchbond Multi-Purpose(ER) -Single Bond Plus (ER) -Clearfil SE Bond (SE) -Scotchbond Universal (SE) + Filtek Bulk Fill Flow	Filtek Z100 (direct resin composite)	Relotec LC	Air-abrasion and phosphoric acid	-	Distilled water at room temperature for at least 24 h	5	µTBS
Sakr [65] (2021)	Human (molar)	Optibond FL (ER)	Filtek Z350XT (indirect resin composite restoration)	-	-	RelyX	Distilled water for 24 h	10	SBS
Sakr [66] (2021)	Human (molar)	Optibond FL (ER)	Filtek Z350XT (indirect resin composite restoration)	-	-	RelyX	Distilled water for 24 h	15	SBS
Gailani et al. [48] (2021)	Human (molar)	-OptiBond FL -OptiBond Universal -Prime and Bond active universal -Scotchbond Universal Adhesive -Future bond Universal single bond -Universal Primer Dual Cured Adhesive -All Bond Universal -Adhese Universal -One coat7 Universal	Lava Ultimate blocks	Telio CS Onlay	Sandblast with cleaning powder	-Maxcem Elite cement -Calibra Ceram Adhesive Resin Cement -Relyx Ultimate Adhesive Resin Cement -Rebilda DC Cement -Duo-Link Universal -Variolink -Solocem cement	Simulated pulpal pressure at room temperature for 24 h	4	μTBS
Deniz et al. [67] (2021)	Human (molar)	-Adper Single Bond 2 (ER) -Single Bond Universal (ER)	-	-	-	RelyX Ultimate Clicker	Distilled water at 37 °C for 24 h	15	SBS
Abo-Alazm et al. [49] (2021)	Human (third molar)	-iBOND (SE) -GLUMA Bond Universal (SE)	Grandio (CAD/CAM resin block)	Cavex	Airborne-particle abrasion (CoJet)	RelyX Unicem	24 h or 6 months of water storage in distilled water	5	μTBS
Abdou et al. [50] (2021)	Bovine (incisor)	-Clearfil Universal Bond Quick -Scotchbond Universal Adhesive -Optibond All-in-one	Katana Avencia Block (CAD/CAM resin block)	Temp bond NE (for multiple-visit)	Alcohol-soaked cotton pellets for 10 s (for multiple visits)	-Panavia V5 -RelyX Ultimate -NX3 Nexus	-	5	μTBS
Oda et al. [47] (2022)	Human (molar)	Clearfil SE Bond 2 (SE) + Clearfil Majesty ES Flow	Katana Avencia Block (CAD/CAM resin blocks)	-	-	-Panavia SA cement plus -Panavia SA cement universal	-	5	μTBS
Guilardi et al. [70] (2022)	Bovine (incisor)	-Single Bond 2 (ER) -Single Bond Universal (SE)	-	Temp-Bond NE	Pumice	-RelyX U200 -Multilink Automix	Distilled water at 37 °C for 24 h	5	μSBS
Nakazawa et al. [51] (2022)	Human (mandibular first molar)	-Clearfil Universal Bond Quick -Clearfil Universal Bond Quick + Clearfil Majesty ES Flow	-Vitablocks Mark II (feldspathic ceramic block)	-	-	-Panavia SA Cement Universal	Cyclic loading for 3 × 105 cycles	16 slabs	μTBS
Pheerarangsikul et al. [68] (2022)	Human (premolar)	-Single Bond Universal (SE/ER) -OptiBond XTR (SE) -Clearfil SE Bond (SE)	Ceram.x SphereTec one (indirect resin composite restoration)	-	Pumice	-RelyX Ultimate -NX3 Nexus -Panavia V5 -Super-Bond C&B	5000 thermocycles	10	SBS
Batista et al. [71] (2022)	Bovine (lower incisor)	-Single Bond Universal (SE) -Single Bond Universal (SE) + Filtek Z350 XT Flow	-	-	Pumice, 37% phosphoric acid	-RelyX Ultimate	24 h or 3 months in distilled water at 37 °C	15	μSBS
Sooksang et al. [52] (2023)	Human (third molar)	-Single Bond Universal (SE) -Optibond FL (ER) (Single and double application)	-	Temp-Bond NE	Pumice	-RelyX U200	Cyclic loading for 50,000 cycles	5 (10 sticks)	μTBS
Kimyai et al. [53] (2023)	Human (third molar)	-All-Bond Universal (SE/ER)	Spectrum (indirect resin composite restoration)	-	-	Bifix SE	7 days at 37 °C or 10,000 thermocycles	30 sticks	μTBS

**Table 3:** Risk of bias.

Study	(I) Teeth Randomization	(II) Materials Used According to Manufacturer’s Instructions	(III) Single Operator	(IV) Sample Size	(V) Standardized sample	(VI) Blinding Operator	VII) Failure Mode	Risk of Bias
Kitasako et al. [72]	N	Y	N	N	Y	N	Y	HIGH
Nikaido et al. [73]	Y	Y	N	N	Y	N	Y	MEDIUM
Jayasooriya et al. [20]	Y	Y	N	N	Y	N	Y	MEDIUM
Nikaido et al. [21]	N	Y	N	N	Y	N	Y	HIGH
Magne et al. [17]	N	Y	N	N	Y	N	Y	HIGH
Islam et al. [22]	Y	Y	N	N	Y	N	Y	MEDIUM
Duarte et al. [74]	Y	Y	N	N	Y	N	Y	MEDIUM
Okuda et al. [23]	Y	Y	N	N	Y	N	Y	MEDIUM
Sultana et al. [24]	Y	Y	N	N	Y	N	Y	MEDIUM
Frankenberger et al. [25]	Y	Y	N	N	Y	N	Y	MEDIUM
Magne et al. [18]	N	Y	N	Y	Y	N	Y	MEDIUM
de Andrade et al. [26]	Y	Y	N	N	Y	N	Y	MEDIUM
Erkut et al. [54]	N	Y	N	N	Y	N	Y	HIGH
Ariyoshi et al. [27]	Y	Y	N	N	Y	N	Y	MEDIUM
Nikaido et al. [28]	N	Y	N	N	Y	N	Y	HIGH
Santos-Daroz et al. [69]	Y	Y	N	N	Y	N	Y	MEDIUM
Kameyama et al. [29]	Y	Y	N	N	Y	N	Y	MEDIUM
Kitayama et al. [30]	Y	Y	N	N	Y	N	Y	MEDIUM
Duarte et al. [19]	N	Y	N	N	Y	N	Y	HIGH
Lee and Park [55]	Y	Y	N	N	Y	N	N	HIGH
Takahashi et al. [31]	Y	Y	N	N	Y	N	Y	MEDIUM
Takahashi et al. [75]	Y	Y	N	N	Y	N	Y	MEDIUM
Feitosa et al. [32]	Y	Y	N	N	Y	N	Y	MEDIUM
Choi and Cho [56]	Y	Y	N	N	Y	N	Y	MEDIUM
Hassan et al. [60]	N	Y	N	N	Y	N	N	HIGH
Sailer et al. [57]	N	Y	N	N	Y	N	Y	HIGH
Dalby et al. [58]	Y	Y	Y	N	Y	N	Y	MEDIUM
Falkensammer et al. [59]	N	Y	N	N	Y	N	Y	HIGH
Duque [33]	Y	Y	Y	Y	Y	N	N	MEDIUM
Giannini et al. [34]	N	Y	N	N	Y	N	Y	HIGH
Santana et al. [35]	Y	Y	N	N	Y	N	Y	MEDIUM
da Silva et al. [36]	Y	Y	N	N	Y	N	Y	MEDIUM
Brigagão et al. [37]	N	N	N	N	Y	N	Y	HIGH
Ishii et al. [38]	N	Y	N	N	Y	N	Y	HIGH
Ferreira-Filho et al. [39]	Y	Y	N	N	Y	N	Y	MEDIUM
Hironaka et al. [40]	N	Y	N	N	Y	N	Y	HIGH
Murata et al. [41]	N	Y	N	N	Y	N	Y	HIGH
Reboul et al. [61]	Y	Y	Y	N	Y	N	Y	MEDIUM
Rigos et al. [62]	Y	Y	Y	N	Y	N	Y	MEDIUM
van den Breemer et al. [42]	Y	N	N	N	Y	N	Y	HIGH
van den Breemer et al. [63]	Y	N	N	N	Y	N	Y	HIGH
Akehashi et al. [43]	Y	Y	N	N	Y	N	Y	MEDIUM
Hayashi et al. [44]	N	Y	N	N	Y	N	Y	HIGH
Sag et al. [64]	Y	Y	N	N	Y	N	N	HIGH
Rozan et al. [45]	Y	Y	N	N	Y	N	Y	MEDIUM
Cesca et al. [76]	Y	Y	N	Y	Y	N	Y	MEDIUM
Carvalho et al. [46]	Y	Y	N	Y	Y	N	Y	MEDIUM
Sakr [65]	Y	Y	N	N	Y	N	N	HIGH
Sakr [66]	Y	Y	N	N	Y	N	N	HIGH
Gailani et al. [48]	Y	Y	N	N	Y	N	Y	MEDIUM
Deniz et al. [67]	Y	Y	Y	Y	Y	Y	Y	LOW
Abo-Alazm et al. [49]	Y	Y	N	Y	Y	N	N	MEDIUM
Abdou et al. [50]	N	Y	N	N	Y	N	Y	HIGH
Oda et al. [47]	Y	N	N	N	Y	N	Y	HIGH
Guilardi et al. [70]	Y	Y	N	Y	Y	N	Y	MEDIUM
Nakazawa et al. [51]	Y	N	N	N	Y	N	Y	HIGH
Pheerarangsikul et al. [68]	Y	Y	N	Y	Y	N	Y	MEDIUM
Batista et al. [71]	Y	Y	N	N	Y	N	Y	MEDIUM
Sooksang et al. [52]	N	N	N	N	Y	N	Y	HIGH
Kimyai et al. [53]	Y	Y	Y	N	Y	N	N	MEDIUM

**Table 4:** IRR values of studies in the systematic review.

	% User Agreement	Kappa	% Data That Are Reliable (through Cohen’s Kappa Test)	Level of Agreement
**(I) Randomization of Teeth**	98.33%	0.9597	92.10%	Almost Perfect
**(II) Manufacturer’s Instructions**	95.00%	0.774	59.985%	Moderate
**(III) Single Operator**	100.00%	1	100%	Almost Perfect
**(IV) Sample Size**	100.00%	1	100%	Almost Perfect
**(V) Standardized Sample**	100.00%	1	100%	Almost Perfect
**(VI) Blinding Operator**	100.00%	1	100%	Almost Perfect
**(VII) Failure Mode**	100.00%	1	100%	Almost Perfect

## Data Availability

Data sharing is not applicable.

## References

[1] Spohr A.M., Borges G.A., Platt J.A. (2013). Thickness of immediate dentin sealing materials and its effect on the fracture load of a reinforced all-ceramic crown. Eur. J. Dent..

[2] Cohen R.G., Razzano M.V. (2006). Immediate dentin sealing using an antibacterial self-etching bonding system. Pract. Proced. Aesthet. Dent..

[3] Pashley E.L., Comer R.W., Simpson M.D., Horner J.A., Pashley D.H., Caughman W.F. (1992). Dentin permeability: Sealing the dentin in crown preparations. Oper. Dent..

[4] Paul S.J., Schärer P. (1997). The dual bonding technique: A modified method to improve adhesive luting procedures. Int. J. Periodontics Restorative Dent..

[5] Nikaido T., Tagami J., Yatani H., Ohkubo C., Nihei T., Koizumi H., Maseki T., Nishiyama Y., Takigawa T., Tsubota Y. (2018). Concept and clinical application of the resin-coating technique for indirect restorations. Dent. Mater. J..

[6] Nikaido T., Inoue G., Takagaki T., Takahashi R., Sadr A., Tagami J. (2015). Resin Coating Technique for Protection of Pulp and Increasing Bonding in Indirect Restoration. Curr. Oral Health Rep..

[7] Dillenburg A.L., Soares C.G., Paranhos M.P., Spohr A.M., Loguercio A.D., Burnett Jr L.H. (2009). Microtensile bond strength of prehybridized dentin: Storage time and surface treatment effects. J. Adhes. Dent..

[8] Magne P. (2005). Immediate dentin sealing: A fundamental procedure for indirect bonded restorations. J. Esthet. Restor. Dent..

[9] Magne P., Nielsen B. (2009). Interactions between impression materials and immediate dentin sealing. J. Prosthet. Dent..

[10] El Mourad A.M. (2018). Assessment of Bonding Effectiveness of Adhesive Materials to Tooth Structure using Bond Strength Test Methods: A Review of Literature. Open Dent. J..

[11] Bertschinger C., Paul S.J., Lüthy H., Schärer P. (1996). Dual application of dentin bonding agents: Its effect on the bond strength. Am. J. Dent..

[12] Paul S.J., Schaerer P. (1997). Effect of provisional cements on the bond strength of various adhesive bonding systems on dentine. J. Oral Rehabil..

[13] Page M., McKenzie J., Bossuyt P., Boutron I., Hoffmann T.C., Mulrow C.D., Shamseer L., Tetzlaff J.M., Akl E.A., Brennan S.E. (2021). The PRISMA 2020 statement: An Updated Guideline for Reporting Systematic Reviews. BMJ.

[14] Hardan L., Devoto W., Bourgi R., Cuevas-Suárez C.E., Lukomska-Szymanska M., Fernández-Barrera M.Á., Cornejo-Ríos E., Monteiro P., Zarow M., Jakubowicz N. (2022). Immediate dentin sealing for adhesive cementation of indirect restorations: A systematic review and meta-analysis. Gels.

[15] Hao J., Lang S., Mante F., Pavelić K., Ozer F. (2021). Antimicrobial and Mechanical Effects of Zeolite Use in Dental Materials: A Systematic Review. Acta Stomatol. Croat..

[16] McHugh M.L. (2012). Interrater reliability: The kappa statistic. Biochem. Medica.

[17] Magne P., Kim T.H., Cascione D., Donovan T.E. (2005). Immediate dentin sealing improves bond strength of indirect restorations. J. Prosthet. Dent..

[18] Magne P., So W.S., Cascione D. (2007). Immediate dentin sealing supports delayed restoration placement. J. Prosthet. Dent..

[19] Duarte S., de Freitas C.R., Saad J.R., Sadan A. (2009). The effect of immediate dentin sealing on the marginal adaptation and bond strengths of total-etch and self-etch adhesives. J. Prosthet. Dent..

[20] Jayasooriya P.R., Pereira P.N., Nikaido T., Tagami J. (2003). Efficacy of a resin coating on bond strengths of resin cement to dentin. J. Esthet. Restor. Dent..

[21] Nikaido T., Nakaoki Y., Ogata M., Foxton R., Tagami J. (2003). The resin-coating technique. Effect of a single-step bonding system on dentin bond strengths. J. Adhes. Dent..

[22] Islam M.R., Takada T., Weerasinghe D.S., Uzzaman M.A., Foxton R.M., Nikaido T., Tagami J. (2006). Effect of resin coating on adhesion of composite crown restoration. Dent. Mater. J..

[23] Okuda M., Nikaido T., Maruoka R., Foxton R.M., Tagami J. (2007). Microtensile bond strengths to cavity floor dentin in indirect composite restorations using resin coating. J. Esthet. Restor. Dent..

[24] Sultana S., Nikaido T., Matin K., Ogata M., Foxton R.M., Tagami J. (2007). Effect of resin coating on dentin bonding of resin cement in Class II cavities. Dent. Mater. J..

[25] Frankenberger R., Lohbauer U., Taschner M., Petschelt A., Nikolaenko S.A. (2007). Adhesive luting revisited: Influence of adhesive, temporary cement, cavity cleaning, and curing mode on internal dentin bond strength. J. Adhes. Dent..

[26] de Andrade O.S., de Goes M.F., Montes M.A. (2007). Marginal adaptation and microtensile bond strength of composite indirect restorations bonded to dentin treated with adhesive and low-viscosity composite. Dent. Mater..

[27] Ariyoshi M., Nikaido T., Foxton R.M., Tagami J. (2008). Microtensile bond strengths of composite cores to pulpal floor dentin with resin coating. Dent. Mater. J..

[28] Nikaido T., Kitasako Y., Burrow M.F., Umino A., Maruoka R., Ikeda M., Tagami J. (2008). Effect of resin coating on dentin bond durability of a resin cement over 1 year. Am J Dent..

[29] Kameyama A., Oishi T., Sugawara T., Hirai Y. (2009). Microtensile bond strength of indirect resin composite to resin-coated dentin: Interaction between diamond bur roughness and coating material. Bull Tokyo Dent Coll..

[30] Kitayama S., Pilecki P., Nasser N.A., Bravis T., Wilson R.F., Nikaido T., Tagami J., Watson T.F., Foxton R.M. (2009). Effect of resin coating on adhesion and microleakage of computer-aided design/computer-aided manufacturing fabricated all-ceramic crowns after occlusal loading: A laboratory study. Eur. J. Oral. Sci..

[31] Takahashi R., Nikaido T., Ariyoshi M., Foxton R.M., Tagami J. (2010). Microtensile bond strengths of a dual-cure resin cement to dentin resin-coated with an all-in-one adhesive system using two curing modes. Dent. Mater. J..

[32] Feitosa V.P., Medina A.D., Puppin-Rontani R.M., Correr-Sobrinho L., Sinhoreti M.A.C. (2010). Effect of resin coat technique on bond strength of indirect restorations after thermal and load cycling. Bull. Tokyo Dent. Coll..

[33] Duque A. (2014). Influence of Immediate Dentin Sealing on the Bond Strength of Indirect Bonded Restorations. Master’s Thesis.

[34] Giannini M., Takagaki T., Bacelar-Sá R., Vermelho P.M., Ambrosano G.M.B., Sadr A., Nikaido T., Tagami J. (2015). Influence of resin coating on bond strength of self-adhesive resin cements to dentin. Dent. Mater. J..

[35] Santana V.B., de Alexandre R.S., Rodrigues J.A., Ely C., Reis A.F. (2016). Effects of Immediate Dentin Sealing and Pulpal Pressure on Resin Cement Bond Strength and Nanoleakage. Oper. Dent..

[36] Da Silva C.J.R., Gonçalves I.C.S., Botelho M.P.J., Guiraldo R.D., Lopes M.B., Júnior A.G. (2016). Interactions between resin-based temporary materials and immediate dentin sealing. Appl. Adhes. Sci..

[37] Brigagão V.C., Barreto L.F.D., Gonçalves K.A.S., Amaral M., Vitti R.P., Neves A.C., Silva-Concílio L.R. (2017). Effect of interim cement application on bond strength between resin cements and dentin: Immediate and delayed dentin sealing. J. Prosthet. Dent..

[38] Ishii N., Maseki T., Nara Y. (2017). Bonding state of metal-free CAD/CAM onlay restoration after cyclic loading with and without immediate dentin sealing. Dent. Mater. J..

[39] Ferreira-Filho R.C., Ely C., Amaral R.C., Rodrigues J.A., Roulet J.F., Cassoni A., Reis A.F. (2018). Effect of Different Adhesive Systems Used for Immediate Dentin Sealing on Bond Strength of a Self-Adhesive Resin Cement to Dentin. Oper. Dent..

[40] Hironaka N.G.L., Ubaldini A.L.M., Sato F., Giannini M., Terada R.S., Pascotto R.C. (2018). Influence of immediate dentin sealing and interim cementation on the adhesion of indirect restorations with dual-polymerizing resin cement. J. Prosthet. Dent..

[41] Murata T., Maseki T., Nara Y. (2018). Effect of immediate dentin sealing applications on bonding of CAD/CAM ceramic onlay restoration. Dent. Mater. J..

[42] van den Breemer C.R.G., Özcan M., Cune M.S., Ayres A.A., Van Meerbeek B., Gresnigt M. (2019). Effect of Immediate Dentin Sealing and Surface Conditioning on the Microtensile Bond Strength of Resin-based Composite to Dentin. Oper. Dent..

[43] Akehashi S., Takahashi R., Nikaido T., Burrow M.F., Tagami J. (2019). Enhancement of dentin bond strength of resin cement using new resin coating materials. Dent. Mater. J..

[44] Hayashi K., Maeno M., Nara Y. (2019). Influence of immediate dentin sealing and temporary restoration on the bonding of CAD/CAM ceramic crown restoration. Dent. Mater. J..

[45] Rozan S., Takahashi R., Nikaido T., Tichy A., Tagami J. (2020). CAD/CAM-fabricated inlay restorations: Can the resin-coating technique improve bond strength and internal adaptation?. Dent. Mater. J..

[46] de Carvalho M.A., Lazari-Carvalho P.C., Polonial I.F., de Souza J.B., Magne P. (2021). Significance of immediate dentin sealing and flowable resin coating reinforcement for unfilled/lightly filled adhesive systems. J. Esthet. Restor. Dent..

[47] Oda Y., Takahashi R., Nikaido T., Tagami J. (2022). Influence of the resin-coating technique on the bonding performance of self-adhesive resin cements in single-visit computer-aided design/computer-aided manufacturing resin restorations. J. Esthet. Restor. Dent..

[48] Gailani H.F.A., Benavides-Reyes C., Bolaños-Carmona M.V., Rosel-Gallardo E., González-Villafranca P., González-López S. (2021). Effect of Two Immediate Dentin Sealing Approaches on Bond Strength of Lava™ CAD/CAM Indirect Restoration. Materials.

[49] Abo-Alazm E.A.E., Safy R.K. (2022). Impact of Immediate Dentin Sealing Using Universal Adhesive under Simulated Pulp Pressure on Microtensile Bond Strength of Indirect Resin Composite Restorations and Dentin Permeability. Eur. J. Dent..

[50] Abdou A., Takahashi R., Saad A., Nozaki K., Nikaido T., Tagami J. (2021). Influence of resin-coating on bond strength of resin cements to dentin and CAD/CAM resin block in single-visit and multiple-visit treatment. Dent. Mater. J..

[51] Nakazawa M., Maeno M., Komoto M., Nara Y. (2022). Appropriate Immediate Dentin Sealing to Improve the Bonding of CAD/CAM Ceramic Crown Restorations. Polymers.

[52] Sooksang O., Wanachantararak S., Sukapattee M. (2023). Effects of single and double application of immediate dentin sealing techniques on marginal leakage and microtensile bond strength of resin cement. J. Int. Dent. Med. Res..

[53] Kimyai S., Bahari M., Abed-Kahnamouei M., Ebrahimi-Chaharom M.E., Asl-Oskouei M.H. (2023). Effect of different application strategies of universal adhesive used for immediate and delayed dentin sealing on the microtensile bond strength of self-adhesive resin cement to dentin with and without aging. J. Clin. Exp. Dent..

[54] Erkut S., Küçükesmen H.C., Eminkahyagil N., Imirzalioglu P., Karabulut E. (2007). Influence of previous provisional cementation on the bond strength between two definitive resin-based luting and dentin bonding agents and human dentin. Oper. Dent..

[55] Lee J.I., Park S.H. (2009). The effect of three variables on shear bond strength when luting a resin inlay to dentin. Oper. Dent..

[56] Choi Y.S., Cho I.H. (2010). An effect of immediate dentin sealing on the shear bond strength of resin cement to porcelain restoration. J. Adv. Prosthodont..

[57] Sailer I., Oendra A.E., Stawarczyk B., Hämmerle C.H. (2012). The effects of desensitizing resin, resin sealing, and provisional cement on the bond strength of dentin luted with self-adhesive and conventional resin cements. J. Prosthet. Dent..

[58] Dalby R., Ellakwa A., Millar B., Martin F.E. (2012). Influence of immediate dentin sealing on the shear bond strength of pressed ceramic luted to dentin with self-etch resin cement. Int. J. Dent..

[59] Falkensammer F., Arnetzl G.V., Wildburger A., Krall C., Freudenthaler J. (2014). Influence of different conditioning methods on immediate and delayed dentin sealing. J. Prosthet. Dent..

[60] Hassan M., Khan T.A., Khan W., Khurram M.S. (2011). The effect of resin coating technique on shear bond strength. Pakistan Oral Dent. J..

[61] Reboul T., Hoang Thaï H.A., Cetik S., Atash R. (2018). Comparison between shear forces applied on the overlay-dental tissue interface using different bonding techniques: An in vitro study. J. Indian. Prosthodont. Soc..

[62] Rigos A.E., Dandoulaki C., Kontonasaki E., Kokoti M., Papadopoulou L., Koidis P. (2019). Effect of Immediate Dentin Sealing on the Bond Strength of Monolithic Zirconia to Human Dentin. Oper. Dent..

[63] van den Breemer C.R.G., Özcan M., Pols M.R., Postema A.R., Cune M.S., Gresnigt M. (2019). Adhesion of resin cement to dentin: Effects of adhesive promoters, immediate dentin sealing strategies, and surface conditioning. Int. J. Esthet. Dent..

[64] Sag B.U., Bektas O.O. (2020). Effect of immediate dentin sealing, bonding technique, and restorative material on the bond strength of indirect restorations. Braz. Dent. Sci..

[65] Sakr O.M. (2021). Immediate Dentin Sealing versus Dentin Air Abrasion Prior to Composite Inlay Luting Procedures. Med. Forum..

[66] Sakr O.M. (2021). Immediate Dentin Sealing Versus Erbium (Er): YAG laser Dentin Ablation Prior to Composite Inlay Luting Procedures. Med. Forum..

[67] Deniz S.T., Oglakci B., Yesilirmak S.O., Dalkilic E.E. (2021). The effect of immediate dentin sealing with chlorhexidine pretreatment on the shear bond strength of dual-cure adhesive cement. Microsc. Res. Tech..

[68] Pheerarangsikul N., Wayakanon P., Wayakanon K. (2022). Effects of Various Functional Monomers on Adhesion Between Immediate Dentin Sealing and Resin Cement. Oper. Dent..

[69] Santos-Daroz C.B., Oliveira M.T., Góes M.F., Nikaido T., Tagami J., Giannini M. (2008). Bond strength of a resin cement to dentin using the resin coating technique. Braz. Oral. Res..

[70] Guilardi L.F., Dapieve K.S., Giordani J.C., Susin A.H., Valandro L.F., Rippe M.P. (2022). Effect of immediate dentin sealing and temporary cement removal on bond strength of resin cements to dentin. Braz. Dent. Sci..

[71] Batista J.M.N., Leite M.M., Sabag M.F., Lopes L.G., Torres É.M. (2022). Influence of the Flowable Resin Layer on Bond Strength Between Resin Cement and a Universal Adhesive Applied in the Immediate Dentin-sealing Technique. Oper. Dent..

[72] Kitasako Y., Burrow M.F., Nikaido T., Tagami J. (2002). Effect of resin-coating technique on dentin tensile bond strengths over 3 years. J. Esthet. Restor. Dent..

[73] Nikaido T., Cho E., Nakajima M., Tashiro H., Toba S., Burrow M.F., Tagami J. (2003). Tensile bond strengths of resin cements to bovine dentin using resin coating. Am. J. Dent..

[74] Duarte R.M., de Goes M.F., Montes M.A. (2006). Effect of time on tensile bond strength of resin cement bonded to dentine and low-viscosity composite. J. Dent..

[75] Takahashi R., Nikaido T., Ariyoshi M., Kitayama S., Sadr A., Foxton R.M., Tagami J. (2010). Thin resin coating by dual-application of all-in-one adhesives improves dentin bond strength of resin cements for indirect restorations. Dent. Mater. J..

[76] Cesca R., Colombo V., Ernst B., Gallo L.M., Özcan M. (2020). Tensile Strength and Failure Types of Direct and Indirect Resin Composite Copings for Perio-Overdentures Luted Using Different Adhesive Cementation Modalities. Materials.

[77] Azad E., Atai M., Zandi M., Shokrollahi P., Solhi L. (2018). Structure-properties relationships in dental adhesives: Effect of initiator, matrix monomer structure, and nano-filler incorporation. Dent. Mater..

[78] Miyazaki M., Ando S., Hinoura K., Onose H., Moore B.K. (1995). Influence of filler addition to bonding agents on shear bond strength to bovine dentin. Dent. Mater..

[79] Van Landuyt K.L., Snauwaert J., De Munck J., Peumans M., Yoshida Y., Poitevin A., Coutinho E., Suzuki K., Lambrechts P., Van Meerbeek B. (2007). Systematic review of the chemical composition of contemporary dental adhesives. Biomaterials.

[80] Eliades G.C., Caputo A.A. (1989). The strength of layering technique in visible light-cured composites. J. Prosthet. Dent..

[81] Rueggeberg F.A., Margeson D.H. (1990). The effect of oxygen inhibition on an unfilled/filled composite system. J. Dent. Res..

[82] Bruzi G., Carvalho A.O., Maia H.P. (2013). Are there combinations of resin liners and impression materials not compatible with IDS technique?. Dent. Mater. J..

[83] Bergmann P., Noack M.J., Roulet J.F. (1991). Marginal adaptation with glass-ceramic inlays adhesively luted with glycerine gel. Quintessence Int..

[84] Ghiggi P.C., Steiger A.K., Marcondes M.L., Mota E.G., Júnior L.H.B., Spohr A.M. (2014). Does immediate dentin sealing influence the polymerization of impression materials?. Eur. J. Dent..

[85] Khakiani M.I., Kumar V., Pandya H.V., Nathani T.I., Verma P., Bhanushali N.V. (2019). Effect of immediate dentin sealing on polymerization of elastomeric materials: An ex vivo randomized controlled trial. Int. J. Clin. Pediatr. Dent..

[86] Özcan M., Barbosa S.H., Melo R.M., Galhano G.A.P., Bottino M.A. (2007). Effect of surface conditioning methods on the microtensile bond strength of resin composite to composite after aging conditions. Dent. Mater..

[87] Stavridakis M.M., Krejci I., Magne P. (2005). Immediate dentin sealing of onlay preparations: Thickness of pre-cured Dentin Bonding Agent and effect of surface cleaning. Oper. Dent..

[88] Ferracane J.L., Stansbury J.W., Burke F.J.T. (2011). Self-adhesive resin cements–chemistry, properties and clinical considerations. J. Oral Rehabil..

[89] Miotti L.L., Follak A.C., Montagner A.F., Pozzobon R.T., Da Silveira B.L., Susin A.H. (2020). Is conventional resin cement adhesive performance to dentin better than self-adhesive? A systematic review and meta-analysis of laboratory studies. Oper. Dent..

[90] Comba A., Paolone G., Baldi A., Vichi A., Goracci C., Bertozzi G., Scotti N. (2022). Effects of Substrate and Cement Shade on the Translucency and Color of CAD/CAM Lithium-Disilicate and Zirconia Ceramic Materials. Polymers.

[91] Udo T., Nikaido T., Ikeda M., Weerasinghe D.S., Harada N., Foxton R.M., Tagami J. (2007). Enhancement of adhesion between resin coating materials and resin cements. Dent. Mater. J..

[92] Gale M.S., Darvell B.W. (1999). Thermal cycling procedures for laboratory testing of dental restorations. J. Dent..

[93] Armstrong S., Breschi L., Özcan M., Pfefferkorn F., Ferrari M., Van Meerbeek B. (2017). Academy of Dental Materials guidance on in vitro testing of dental composite bonding effectiveness to dentin/enamel using micro-tensile bond strength (μTBS) approach. Dent. Mater..

